# Association between serum ferritin level, cardiac and hepatic T2-star MRI in patients with major β-thalassemia

**Published:** 2014-03-15

**Authors:** A Eghbali, H Taherahmadi, M Shahbazi, B Bagheri, L Ebrahimi

**Affiliations:** 1 Pediatric Hematologist & Oncologist, Department of Pediatrics, Arak University of Medical Sciences, Arak- Iran; 2 Department of Pediatrics, Arak University of Medical Sciences, Arak- Iran; 3 Student of Medicine, Arak University of Medical Sciences, Arak- Iran; 4 Pharmacologist, Department of pharmacology, Semnan University of Medical Sciences, Semnan-Iran; 5 Blood transfusion research center, High Institute for Research and Education in Transfusion Medicine, Tehran-Iran

**Keywords:** Ferritin, Iron chelator, Major thalassemia, T2*MRI

## Abstract

**Background:**

Frequent blood transfusion is often associated with iron overload. Proper use of iron chelators to treat iron overload requires an accurate measurement of iron levels. Magnetic resonance T2-star (T2* MRI) can measure iron level in the heart and liver. Our goal was to see whether an association exists between serum ferritin level and T2* MRI in patients with major beta thalassemia.

**Materials and Methods:**

Sixty patients with a diagnosis of major beta thalassemia were enrolled in the study. They were older than five years old and needed regular transfusion.

Cardiac and hepatic T2*MRI and mean serum ferritin levels were measured within 3 months.

**Results:**

No significant correlation was observed between serum ferritin level and cardiac T2*MRI (p=0.361, r=-0.120).However, a significant correlation was observed between serum ferritin and liver T2*MRI (p=0.021, r=-0.297).

**Conclusion:**

Our results showed an association between hepatic T2*MRI and serum ferritin level.

## Introduction

Thalassemia is the most common genetic disorder in the world (1). Frequent blood transfusion as a part of major thalassemia treatment is often linked to iron overload that can be deposited in many organs especially in the heart and liver. Heart failure secondary to iron over load is the main cause of death in patients with major thalassemia. When heart failure develops, the prognosis is usually poor. Heart failure estimated as 70% cause of mortality in major thalassemia. Iron chelators are routinely used to treat iron overload. For correct use of these drugs, an accurate measurement of iron levels is necessary. Several methods are applied to measure iron levels. Measurement of serum ferritin is relatively a reliable test but is not valuable in the liver involvement and elderly; moreover, infections and hepatic diseases can cause false increase in the level of serum ferritin. The most accurate method to measure iron level is liver biopsy; however, it is invasive and unable to provide an accurate measurement of heart iron level (2,3). During recent years, non-invasive methods have gained prominence. Magnetic resonance T2-star (T2* MRI) is a method for assessment of high molecular weight iron complexes induced T2 relaxation enhancement like ferritin and hemosiderin. (4-16).T2* MRI can measure iron levels in the heart and liver and is helpful for early diagnosis of the myocardial hemosiderosis before the initiation of clinical manifestations.The present study was designed to find association between serum ferritin and T2* MRI of the heart and liver in patients with major thalassemia.

## Materials and Methods

This was a cross- sectional descriptive study during 2011 to 2012. A total of 60 patients with a diagnosis of major beta thalassemia admitted to Amir Kabir Hospital, Arak, Iran, were enrolled in the investigation. They were older than five years old and required regular transfusion. Demographic data, mean level of serum ferritin during last 3 months and T2*MRI results were recorded in a questionnaire. The sampling was performed on the basis of simple random method. The exclusion criteria were as follows: Patients younger than 5 years old, advanced hepatic and cardiac disease and patients with hepatitis B or C. The ethical board of the university approved the study and informed consent letter was taken from participants.

T2*MRI

MRI was performed by Magneto Symphony Graniand 32, 1.5 Tesla (Siemens, Germany, 2003) in Noor Clinic (Tehran, Iran). Each scan lasted about 30 minutes and included the measurement of hepatic and cardiac T2*. Cut off points in this MRI instrument are as follows: Liver: normal > 6.3ms, mild: 2.8-6.3ms, moderate: 1.4-2.7ms, severe <1.4ms. Cardiac: normal >20ms, mild: 14-20ms, moderate: 10-14ms, severe <10ms. 

**Measurement of serum levels of ferritin**

Serum ferritin level was measured by ELISA (Awareness technology, US).The findings of MRI and serum ferritin were assessed contemporarily. Then mean of serum ferritin level was recorded during last 3 months. It was measured in patients who showed no evidence of bacterial or viral infection.

**Statistical analysis**

Data are presented as mean±SD. Then mean of serum ferritin level was recorded during last 3 months. Linear regression and other descriptive statistic tests were used. Statistical significance was considered as p<0.05. All of analyses were performed using SPSS software version 16.

## Results

The study was performed on 60 patients with major beta thalassemia (55% male and 45% female). The mean of age of patients was 17.65 ± 9.28 years and its range was 5 to 50. Hemogolbin values ranged from 6.7 to 11.5 (mg/dl) before transfusion. Mean level of hemoglobin was 9.1 (mg/dl) before transfusion. Mean age of transfusion was 1.1 ± 0.8. The mean of serum ferritin level was 1927.1 ng /dl (103.2-11300 ng /dl). The mean of cardiac T2* was 26.46 ± 9.19ms (8.36-45.8) and the mean of hepatic T2* was 5.30± 4.29 ms (0.95-19.72). . Four patients underwent splenectomy. Hepatic T2*MRI is presented in Table 1 and Graph1 and Cardiac T2*MRI is shown in Table 2 and Graph 2. A significant correlation was observed between serum ferritin and liver T2*MRI (p=0.021, r=-0.297). No significant correlation was seen between age and rate of blood transfusion and also between serum ferritin and cardiac T2*MRI (p=0.361, r=-0.120)

**Table I:** Relationship between Serum levels of ferritin and hepatic T2*MRI

**Number of patients Age (year) **	** Serum Ferritin **		Hepatic T2*MRI
14 (23.3%) 14.35± 7.27	1260± 235		normal>6.3ms
28 (46.7%) 18.25± 9.47	1655± 388		mild6.3-2.8 ms
15 (25%) 17.53± 7.32	2338± 449		Moderate2.8-1.4ms
3 (5%) 28± 19.15	5510± 1555		severe<1.4ms

Data are presented as mean ± SD or number (%)

**Table II:** Serum levels of ferritin and cardiac T2*MRI

**Number of patients Age (year)**	** Serum ferritin **		Cardiac T2*MRI
46 (76.7%) 19.5± 9.5	1913± 305		Normal>20ms
9 (15%) 11.3± 5	1987± 342		Mild14-20 ms
3 (5%) 8.6± 2.5	666± 100		moderate10-14ms
2 (3%) 17± 7	3849± 289		Severe<10ms

Data are presented as mean ± SD or number (%).

**Figure 1:**
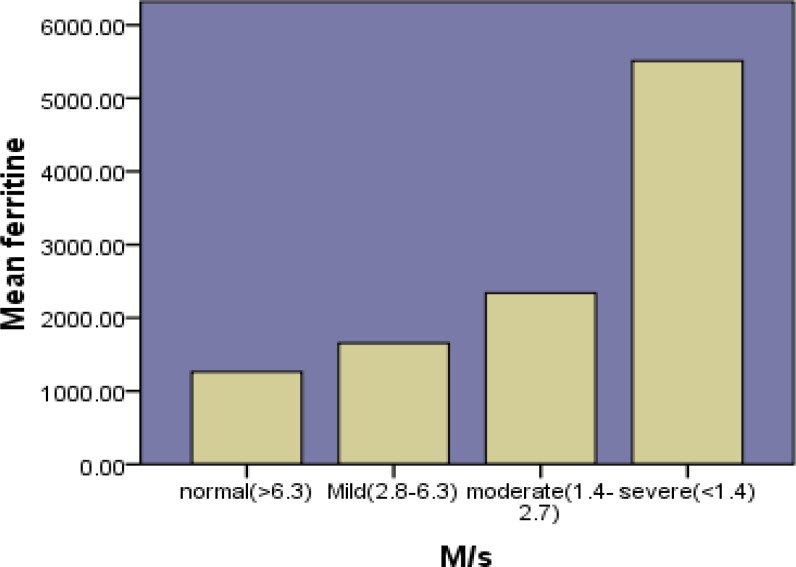
Serum levels of ferritin and hepatic T2*MRI

**Figure 2:**
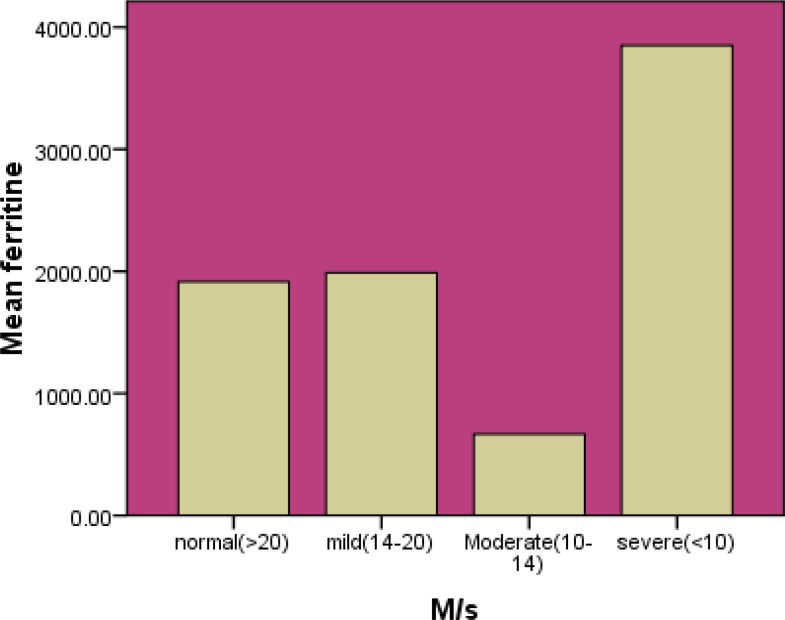
Serum levels of ferritin and cardiac T2*MRI

## Discussion

There are several methods to measure iron levels in different organs. Liver disease and inflammatory responses can increase amount of serum ferritin, so its measurement is not very reliable. Liver biopsy is the gold standard method to measure iron level but it is invasive and cannot predict iron level of the heart. Echocardiography is another method, which is only reliable in advanced iron overloading. In the present study, T2*MRI was used for to measurement of iron levels in the heart and liver. It’s found that cardiac T2*MRI is associated with the mean of age. Moreover, the result showed that younger patients had higher levels of hepatic T2*MRI. Shamsian’s study showed that there was a direct association between cardiac T2*MRI and mean of age. However, two similar studies proved a reverse association between T2*MRI and mean of age (17). Surekha Tony and colleague could not show a significant association between cardiac T2*MRI and serum ferritin(18). In concert with these observations, Alberto et al demonstrated that measurement of serum ferritin did not have a prognostic value (19). In our study, correlation between age and cardiac T2*MRI was direct which can be caused by sensitivity of T2*MRI in detecting cardiac ironload. In study of shamsian and et al in mofid children`s hospital in 2011, similar results were obtained but opposite results were seen in Perifanis study in 2007 (17). In our study correlation between age and hepatic T2*MRI was reverse. These results were matched with chiristoforidis study in Greece (20). This study reported that hepatic T2*MRI had strong correlation with serum ferritin and could be used to estimate iron levels of the body. 

Zamani`s study revealed no reasonable correlation between histological grade of siderosis (HGS) and serum ferritin. A moderate correlation was seen between serum ferritin levels and hepatic T2* levels. Iron concentration of the liver showed significant correlation with hepatic T2*. These results indicated that T2* MRI measurement is of more value than HGS in patients with thalassemia (21).

## Conclusion

Our results showed an association between hepatic T2*MRI and serum ferritin. It is suggested to use this method in order to have an accurate measurement of iron level in patients with thalassemia. We can recommend hepatic and cardiac T2*MRI in addition to measurement of serum ferritin for better evaluation of patients with major thalassemia.

## Conflict of interest

The authors have no conflict of interest.
